# Overview of Colistin Resistance in the Middle East and North Africa (MENA) Region

**DOI:** 10.3390/microorganisms14081693

**Published:** 2026-08-01

**Authors:** Rami Saniour, Rita-Nour Awad, Ali Khalouf, Liza Dib, Bassem Derbas, Charbel Al-Bayssari

**Affiliations:** 1 Faculty of Medicine and Medical Sciences, University of Balamand, Kalhat, Tripoli P.O. Box 100, Lebanon; 2 Department of Medical Laboratory Sciences, Faculty of Health Sciences, University of Balamand, Kalhat, Tripoli P.O. Box 100, Lebanon

**Keywords:** colistin resistance, multi-drug resistance, gram-negative bacteria-middle east, North Africa

## Abstract

Colistin (polymyxin E), a last-resort antibiotic for the treatment of multidrug-resistant Gram-negative bacterial infections, is becoming increasingly compromised by the rapid emergence and dissemination of resistance. The extensive use of colistin in agriculture and veterinary medicine has accelerated the spread of plasmid-mediated mobile colistin resistance (mcr) genes, facilitating transmission across human, animal, and environmental reservoirs. This review provides a comprehensive overview of the epidemiology, molecular mechanisms, and geographical distribution of colistin resistance in the Middle East and North Africa (MENA) region. Analysis of the available literature indicates that Tunisia reports the highest prevalence of colistin-resistant Gram-negative bacteria in North Africa (58.09%), followed by Egypt (24.76%), Algeria (14.29%), and Libya (2.86%). Across the region, *mcr-1* is the predominant plasmid-mediated resistance determinant, whereas chromosomal alterations involving the *mgrB*, *pmrA/pmrB*, and phoP/phoQ regulatory systems are the principal non-plasmid-mediated mechanisms. Resistance has been documented in major clinical pathogens, including *Escherichia coli*, *Klebsiella pneumoniae*, *Acinetobacter baumannii*, and *Pseudomonas aeruginosa*, as well as in livestock, poultry, aquaculture, wildlife, food products, and environmental samples, highlighting the interconnected nature of resistance transmission under a One Health framework. The evidence demonstrates substantial geographical variability and significant gaps in surveillance across several MENA countries, limiting accurate regional burden estimates. Strengthening antimicrobial stewardship, harmonized surveillance programs, molecular monitoring of resistance determinants, and the implementation of One Health strategies are essential to limit the continued emergence and dissemination of colistin resistance throughout the region.

## 1. Introduction

Since its discovery in 1947, colistin has been a key antibiotic, especially against multidrug-resistant Gram-negative bacteria (GNB) [1]. It remains one of the few effective treatment options for infections caused by carbapenem-resistant isolates of *Escherichia coli*, *Klebsiella pneumoniae*, and *Acinetobacter baumannii*, underscoring its vital role in modern healthcare [1]. However, extensive and prolonged use of colistin, particularly in agricultural and veterinary practices, has contributed to a worrying global rise in resistance to this last-resort antibiotic [2].

The increasing prevalence of colistin-resistant bacteria poses a major global public health challenge. These resistant strains are especially common in areas with high colistin use, such as in livestock farming, and in regions with limited healthcare infrastructure, including conflict-affected zones. International health organizations have urged swift action in response to this escalating risk, including improved antimicrobial stewardship and restrictions on colistin use in non-human settings [2].

The MENA region has not been spared this growing crisis. In recent years, several studies have documented the presence of colistin-resistant bacteria in clinical, agricultural, and environmental settings across countries including Egypt, Tunisia, and Algeria. The rise in colistin resistance in these regions is closely linked to the high use of antibiotics in agriculture, poor regulatory enforcement, and limited access to alternative treatments [3,4,5]. In refugee camps and underserved communities, where overcrowding and inadequate sanitation create ideal conditions for the spread of resistant bacteria, the situation is even more dire [6].

With the global surge in colistin-resistant bacteria and the widespread prevalence of resistance genes such as *mcr-1*, it is crucial to implement coordinated antimicrobial stewardship programs that address problems of colistin resistance in both animal and human health. These initiatives must be reinforced with improved surveillance, particularly in regions with high antibiotic usage and limited healthcare infrastructure, to curb the further spread of resistance.

This review aims to provide a comprehensive overview of the epidemiology of colistin resistance in the MENA region, examining its prevalence across human, animal, and environmental sectors. By analyzing the sources and patterns of the spread of resistance, this review seeks to contribute to the development of targeted interventions aimed at mitigating this escalating threat to public health.

## 2. Overview of Colistin Resistance Mechanisms

Koyama et al. discovered colistin, a historical polypeptide antibiotic belonging to group E, from cultures of *Paenibacillus polymyxa* subspecies *colistinus* [1]. With its bactericidal qualities, colistin primarily affects GNB and is ineffective against anaerobic, Gram-positive, and Mycoplasma bacteria [7]. Interaction with lipopolysaccharide (LPS), a crucial part of GNB outer membrane, is its principal method of action [8]. More specifically, the negatively charged lipid A part of LPS interacts with divalent cations, mainly Mg^2+^ and Ca^2+^, to help stabilize the outer membrane [9]. Because of its net positive charge, colistin binds to LPS very strongly. This causes membrane disruption and the eventual release of LPS by electrostatically displacing cations [8]. The introduction of colistin into the outer membrane is made possible by its lipophilic, acid-fat chain, which also disrupts membrane permeability and makes the inner membrane easier to access. Leakage of cellular contents and bacterial mortality are caused by disruptions in the integrity of the inner membrane. Although this process is widely acknowledged to be the basic mechanism of the antibacterial activity of colistin, the specific pathways leading to the death of bacterial cells are still not fully known [10].

The understanding of the action of colistin and fundamental resistance mechanisms has been aided by research on strains that are naturally resistant to it. Certain *Enterobacteriaceae* species, such as *Proteus* and *Serratia marcescens*, frequently exhibit resistance to polymyxins [11]. The lipid compositions of *Proteus* strains that are sensitive to polymyxin B and those that are resistant to it are essentially identical, indicating that resistance is not caused by the lipids themselves but rather by the lipid composition of the cell envelope [11]. Research in the field of antibiotic resistance has revealed a number of enzymes that can block the action of colistin. Initially, the colistinase enzyme of *Pseudomonas polymyxa* was identified as a serine protease. By precisely cleaving a peptide bond between the side tripeptide and the heptapeptide ring of the colistin molecule, this enzyme can break down the protein [11]. Apr protease is another polymyxin inhibitory enzyme that has been found in *Bacillus licheniformis*. This alkaline protease breaks down colistin at two different sites: between the heptapeptide ring and the tripeptide side chain and between L-threonine and L-diaminobutyric acid within the heptapeptide ring [12]. Furthermore, recent research has shown that *Pseudomonas aeruginosa* possesses N-acetyltransferases that are able to acetylate polymyxins B and E. By adding an acetyl group, these enzymes alter polymyxin, although it is unclear how this alteration affects colistin resistance [13]. These findings pave the way for future investigations into the molecular mechanisms underlying the inactivation of colistin by these enzymes and the creation of novel approaches to creating antibiotic-resistance-fighting therapeutics.

Two primary categories of mechanisms have emerged in the fight against colistin resistance. The first is the mechanism of acquired resistance, which involves the horizontal transfer of resistance genes. The second is the intrinsic mechanism of resistance, which encompasses genetic changes and cellular modifications that enable bacterial survival against this critical antibiotic.

### 2.1. Acquisition of Resistance

Combatting colistin resistance is a major concern, since resistance genes can be acquired by horizontal gene transfer. Prominent examples include the phosphoethanolamine transferase (PEtN) gene (*mcr-1* to *mcr-10*). Lipid A, the lipopolysaccharide (LPS) targeted by colistin, has its structure modified by these enzymes, which reduces the binding affinity of the antibiotic [14]. Much of the dissemination of the *mcr* gene in bacterial populations arises from its common presence on plasmids and other mobile genetic elements. In other words, bacteria can rapidly transform, allowing resistance to spread both within and between bacterial species [14,15].

**Figure 1:**
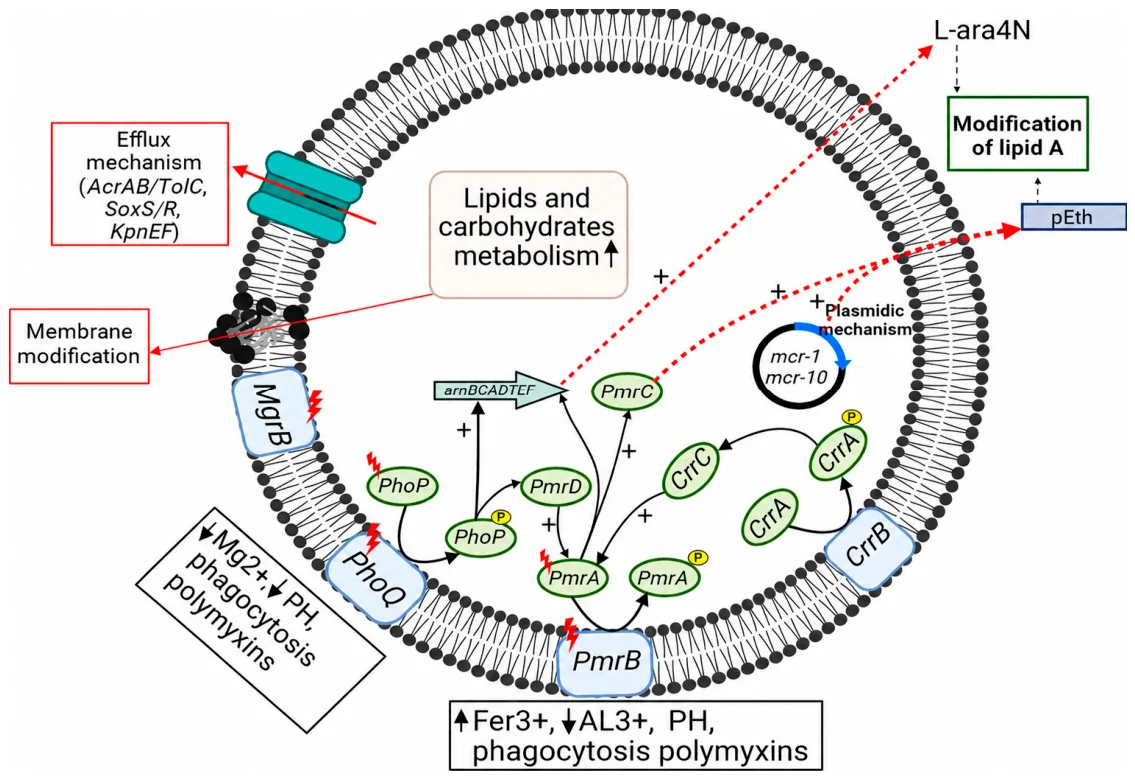
Mechanisms of colistin resistance: membrane modifications, efflux systems, and lipid A alterations. MgrB: MgrB regulator protein (PhoP–PhoQ system regulator); PhoP: Phosphate regulon response regulator PhoP; PhoQ: Phosphate regulon sensor kinase PhoQ; PmrA: Polymyxin resistance response regulator A; PmrB: Polymyxin resistance sensor kinase B; PmrC (EptA): Phosphoethanolamine transferase (EptA); PmrD: PmrD connector protein; CrrA: Colistin resistance regulator A; CrrB: Colistin resistance regulator B; CrrC: Colistin resistance regulatory protein C; arnBCADTEF: arnBCADTEF operon; mcr-1–mcr-10: Mobile colistin resistance genes 1–10; L-Ara4N: 4-amino-4-deoxy-L-arabinose; pEtN: Phosphoethanolamine; AcrAB/TolC: AcrAB-TolC efflux pump; SoxS/R: SoxS/SoxR oxidative stress regulon; KpnEF: KpnEF efflux pump; Mg^2+^: Magnesium ion; Fe^3+^: Ferric iron; Al^3+^: Aluminum ion.

Furthermore, the inclusion of the *mcr* gene on plasmids adds another layer of complexity to the fight against resistance, as plasmids have the ability to contain numerous resistance genes that simultaneously confer resistance to multiple antibiotics. Co-resistance, also known as multidrug resistance, is a phenomenon that drastically lowers the number of therapies that are accessible and increases the difficulty of treating illnesses brought on by bacteria that have developed resistance [14] (Figure 1).

### 2.2. Intrinsic Mechanisms of Resistance to Colistin

Colistin resistance is a complex phenomenon that is frequently caused by changes in the composition and structure of bacterial cell membranes, namely the lipid A component of lipopolysaccharides (LPS) [14]. Genetic variations are often the cause of these changes, which can make bacteria less sensitive to the action of colistin.

#### 2.2.1. PhoP/PhoQ Two-Component System

One of the most important two-component systems in colistin resistance is PhoP/PhoQ. This was first discovered to control the activation of many genes related to virulence, survival, and the accumulation of resistance of *S. typhimurium* to the action of cationic antimicrobial peptides (CAMPs), also known as *pag* genes (polymyxin-activated genes). The PhoQ protein has been proposed to function as a membrane-associated protein kinase, phosphorylating PhoP in response to external signals and thereby activating *pag* gene promoters. This system interferes with the transcription of more than 40 *Salmonella* genes in response to polymyxin B and restricted doses of Mg^2+^ and other divalent cations, thereby inducing virulence. After an examination of these different genes regulated by PhoP, it has been concluded that this PhoP/PhoQ regulator controls pathogenicity in a variety of bacterial species, aids in adapting to restrictive Mg^2+^ conditions, controls the alteration of many components of the cell envelope, and regulates several species-specific genes. By including aminoarabinose and 2-hydroxy myristate via extraction of lipid A and LPS from various *S. typhimurium* strains, the PhoP/PhoQ system further controls LPS alterations [2,14] (Figure 1).

#### 2.2.2. The PmrAB Two-Component System

One of the major mechanisms contributing to colistin resistance is the activation of the PmrAB two-component regulatory system, which induces modifications of lipid A in the outer membrane and reduces colistin binding. This system regulates the expression of genes that modify LPS and was initially discovered in polymyxin-resistant mutants. This process is primarily driven by the pmrCAB operon, which produces the enzymes necessary for LPS modification. Changes to the *pmrA* gene in this system could lead to a reduction in the binding of colistin to LPS molecules, which would decrease the ability of the antibiotic to burst bacterial membranes. Furthermore, PmrA and PmrB form a two-component system (TCS), and PmrC, also known as pagB, appears to be involved in LPS modification.

The PmrAB system is known to regulate approximately 20 verified genes, with up to 100 genes in *Salmonella*, based on microarray, mutagenesis, and in silico analyses. Orthologs of these genes can be found in various Gram-negative pathogens. While the PmrAB system is primarily involved in cell-surface covalent changes through the addition of Ara4N and pEtN to LPS, it also responds directly to unusual environmental conditions such as high Fe^3+^. PhoPQ indirectly activates the PmrAB system via the LPS protein PmrD [2,14,15] (Figure 1).

#### 2.2.3. The mgrB Gene as a Regulator of the PhoP/PhoQ Pathway

The PhoP/PhoQ two-component system is activated under low extracellular magnesium conditions [16]. More research revealed that the PhoP protein directly binds to the *mgrB* promoter region in response to Mg^2+^ ions. Additionally, PhoP activates the *mgrB* gene, also known as yobG, as shown by transcriptional studies and microarray analysis used to discover the gene in *Salmonella enterica* [17]. According to this, *mgrB* is an essential regulator of the PhoP/PhoQ pathway, which operates during stress responses by feedback inhibition [18,19] (Figure 1).

#### 2.2.4. Coordination with crrAB Genes

Along with the PhoP/PhoQ and PmrAB systems, the *crrAB* genes play a role in coordinating bacterial responses to colistin and other antimicrobials. These genes modulate bacterial sensitivity to antibiotics by interacting with the aforementioned resistance mechanisms [14,20,21]. To fully grasp the range of processes underlying colistin resistance, it is imperative to comprehend the interactions among different regulatory pathways (Figure 1).

#### 2.2.5. Various Mechanisms of Resistance

In addition to their regulatory systems, bacteria employ a variety of techniques to evade the effects of colistin. These include the hyperexpression of efflux pumps (AcrAB-TolC, SoxS/R, KpnEF), which actively extract colistin from bacterial cells to lower its intracellular concentration and potency, and protective capsules, which serve as physical barriers to stop antibiotic penetration [7] (Figure 1).

## 3. Colistin Resistance in Humans in the MENA Region

**Figure 2:**
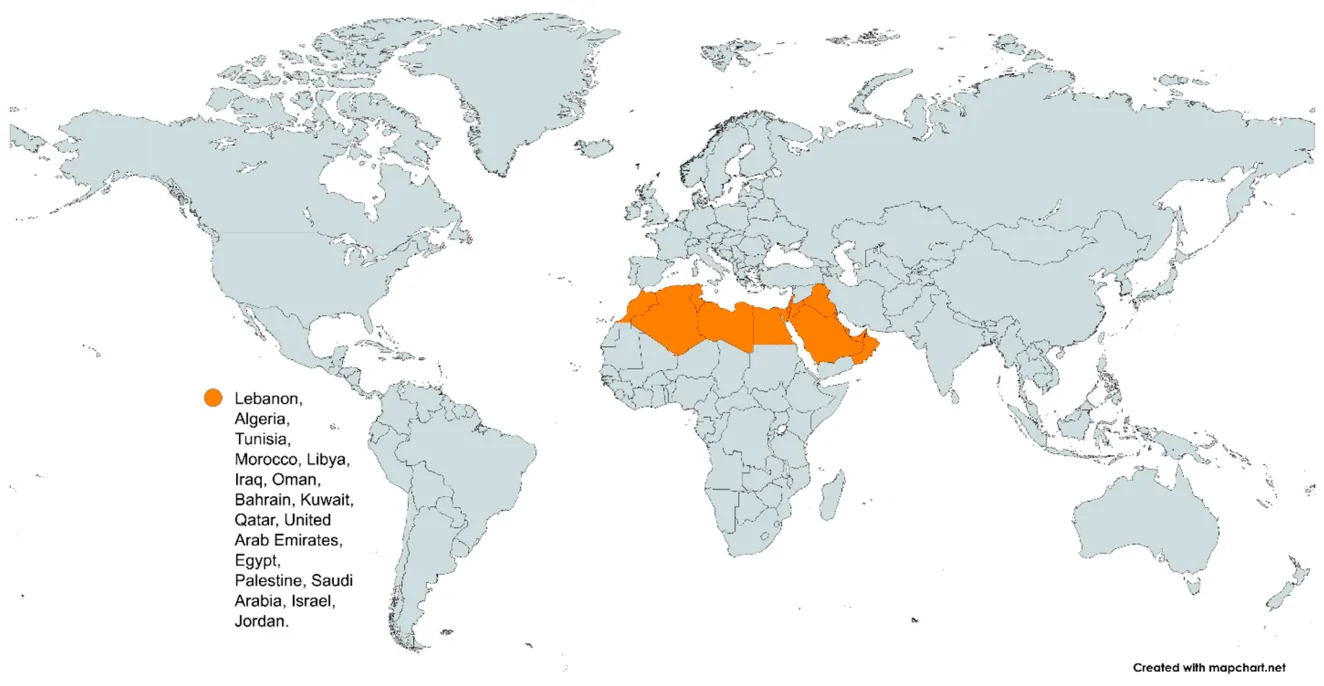
Global distribution of colistin resistance genes in humans: spotlight on the MENA region.

The use of colistin is on the rise around the world, representing a potential risk to human health. Firstly, due to the elevated level of Gram-resistance, especially *Enterobacteriaceae*, *P. aeruginosa*, and *A. baumannii*, and secondly due to its application across several domains, including fish farming and livestock nutrition, which can be transferred along the food chain to healthy humans. In one recently published paper, researchers reported that Tunisia showed the highest prevalence of colistin-resistant GNB in North Africa (58.09%), followed by Egypt (24.76%), Algeria (14.29%), and Libya (2.86%) [22]. According to a systematic review that collected available data on colistin resistance in all 54 African countries in 2020, Olowo-Oker et al. concluded that North Africa reported the most isolates with *mcr* genes, primarily *mcr-1*, *mcr-2*, *mcr-3*, and *mcr-8*. Their analysis suggested that these findings could be attributed to increased colistin use in African agriculture and the proximity of North Africa to Europe, resulting in the importation of resistant bacteria, especially *Escherichia coli* and *Klebsiella pneumoniae* [23]. In a more recent systematic review that targeted carbapenem and polymyxin resistance, Venne et al. studied polymyxin resistance in *E. coli* and *Klebsiella* isolates in detail, from 2010 until 2023 in each African country. Their findings indicated low rates of *E. coli* and *Klebsiella* polymyxin resistance in Algeria, Egypt, Morocco, and Tunisia. *E. coli* isolates from Sudan showed no polymyxin resistance, while *Klebsiella* isolates exhibited low polymyxin resistance. As for Sudan, both isolates were insufficient, and no definitive conclusions were drawn. In the second part of their review, Venne et al. discussed the different genotypic determinants of polymyxin resistance in *E*. *coli*, describing *mcr-1* in Algeria, Egypt, and Sudan, *mcr-8* in Algeria, *mgrB* mutations in Egypt, Libya, and Tunisia, and *phoPQ/pmrAB* mutations in Algeria, Egypt, and Tunisia [24] (Figure 2).

### 3.1. Colistin Resistance in Algeria

The earliest data on colistin resistance in Algeria dates back to 2011, when Berrazeg et al. reported the first case of *mcr-mediated* colistin resistance in a clinical *E. coli* isolate obtained in 2011 from Algeria [25]. In a paper focusing on another GNB, *A. baumannii*, Bakour et al. studied the susceptibility of 12 different isolates to carbapenems and colistin from patients hospitalized between October 2013 and March 2014 in the Beni Messous University Hospital in Algiers. The results revealed a single colistin-resistant *A. baumannii* strain with a Minimum Inhibitory Concentration (MIC) of 16 mg/L. This was the first colistin-resistant carbapenemase-producing *A. baumannii* clinical isolate reported in Algeria. Further molecular analysis demonstrated the presence of a mutated *pmrB* gene responsible for the resistance mechanism. As previously discussed, the majority of studies conducted on a global scale as well as in Algeria have indicated increased resistance to colistin due to its effectiveness in the treatment of infections associated with multidrug-resistant (MDR) bacteria [26]. However, other studies have reported the presence of colistin resistance in people who have not been given the drug before [25]. From this point onwards, researchers shifted their focus to the different factors contributing to colistin resistance in Algeria, including veterinary medicines and agriculture [27]. In a more recent study, ready-to-eat (RTE) foods were considered to be a major source of resistant bacteria, causing many foodborne diseases. In their research, Zaatout et al. collected 204 different samples of RTE foods (cream-filled pastries, salads, and soups) from four provinces in Algeria and used simplex polymerase chain reaction (PCR) to screen for the *mcr-1* gene as well as multiplex PCR for the ESBL/AmpC genes. For the first time, the results identified colistin-resistant ESBL-producing Enterobacterales strains carrying the *mcr-1* gene in RTE foods in Algeria, with 12.5% of samples (6:48) being colistin-resistant [28].

### 3.2. Colistin Resistance in Tunisia

According to Mezghani Maalej et al., the first colistin-resistant bacteria were reported in Tunisia in 2005. In a retrospective study conducted at the Habib Bourguiba and Hedi Chaker Teaching Hospital in Sfax, Tunisia, a total of 121 strains of colistin-resistant *Enterobacteriaceae* were collected from 93 patients. The study revealed that *Enterobacter cloacae* had the highest rate (1.5%) of colistin resistance, followed by *K. pneumoniae* with 1.2%, and *E. coli* with 0.09%. In addition, the study showed that 86% of the isolated strains had homogeneous colistin resistance (MIC between 4 mg/L and ≥1024 mg/L), while the remaining 14% had heterogeneous colistin resistance (MIC ≥ 1024 mg/L), which was observed mainly in *E. cloacae* strains [29]. Furthermore, a retrospective study that was conducted between June 2015 and March 2016 on a total of 940 *Enterobacteriaceae* isolates from different wards of the Tahar Sfar University Hospital aimed to determine the prevalence and mechanisms of colistin and carbapenem resistance among a collection of carbapenemase-resistant *K. pneumoniae* isolates. Using S1 nuclease pulsed-field gel electrophoresis (S1- PFGE), Southern blotting, and PCR-based replicon typing, this study showed that 24.1% of the carbapenem-resistant *K. pneumoniae* isolates were resistant to colistin (MIC: 12–24 μg/mL), which is primarily due to the inactivation of the *mgrB* gene by an insertion or mutation in its promoter or coding sequence [30]. In a separate study that focused on clonal spread of colistin-resistant *K. pneumoniae* coproducing KPC and VIM carbapenemases in Neonates at Rabta Teaching Hospital of Tunis (Tunisia) from 1 January 2014 to 31 December 2014, 21 isolated samples were collected from 19 patients hospitalized in the neonatal unit and two patients hospitalized in the adult intensive care unit (ICU). Data revealed that all the *K. pneumoniae* isolated strains were resistant to colistin with MICs of between 8 mg/L and 12 mg/L. The authors concluded that in 2014, the frequency of colistin-resistant *K. pneumoniae* was 2.96% [31]. An additional retrospective analysis was carried out by Tanfous et al. focusing on the resistance determinants of *K. pneumoniae* to colistin. Isolated samples from immunocompromised patients in the National Center for Bone Marrow Transplantation in Tunisia were limited, and the *mcr-1* gene, which was negative, was the only one studied. However, reported results showed that 3.4% of *K. pneumoniae* isolates exhibited resistance to colistin [32]. In their study, Jaidane et al. focused on two carbapenem-resistant *A. baumannii* isolates, AbS1 and AbS2, isolated from the University Hospital of Sahloul in Sousse, Tunisia. Using disk diffusion antibiograms, the researchers found that AbS1 was susceptible to colistin, while AbS2 was resistant to it, due to a disruption of the binding site on the bacterial membrane by the *pmrA* and/or *pmrB* mutation or loss of LPS by lipid A biosynthesis gene mutations [33]. Additionally, a recent multicenter prospective study [34] on colistin-resistant Gram-negative bacilli isolated 836 GNB strains mainly from urine, blood, and sputum, from six Tunisian university hospitals during a two-month period (March–April 2019). In this study, Ferjani et al. used a multiplex PCR assay to identify the presence of MCR genes (*mcr-1* to *mcr-9*) in all colistin-resistant isolates. Of all the isolates, 5% showed resistance to colistin, mainly *P. aeruginosa* (9.3%), *E. coli* (7%), and *K. pmeumoniae* (2.7%). In addition, four of these *E. coli* isolates had the *mcr-1* gene with a MIC of between 16 mg/L and 32 mg/L [34].

### 3.3. Colistin Resistance in Morocco

Although there is limited data available about colistin resistance in humans in Morocco, in one cohort study, Leangapichart et al. screened for the presence of the *mcr-1* gene in pilgrims traveling to Mecca and returning to their home country between 19 September and 12 October 2024. Two out of the 440 rectal swab samples were from Moroccans, and both showed a positive PCR for *mcr-1-producing E. coli*, each with a unique ST/CC and antimicrobial resistance pattern (1. ST/CC: ST602/CC446, ATB resistance pattern: AMX-AMC-SXT-2. ST1300c, ST1300c). Despite inadequate data about colistin resistance in humans in Morocco, this cohort study drew attention to an important issue: the contribution of travelers to importing the mobile colistin-resistant plasmid to different countries [35].

### 3.4. Colistin Resistance in Libya

The first documentation of colistin resistance in Libya dates to a study that was conducted between 2014 and 2015, when Kieffer et al. collected a total of 28 *A. baumannii* and 33 *Klebsiella* spp. isolates from patients at the Tripoli Medical Center in Libya. Using the Rapid PolymyxinTM NP test, five out of 28 *A. baumannii* isolates and six out of 33 *Klebsiella* isolates showed colistin resistance with MICs of between 4 μg/mL and 128 µg/mL and MICs of between 64 μg/mL and 128 µg/mL, respectively [36]. Venne et al. further investigated the mechanism of resistance of *A. baumannii* and *Klebsiella* isolates, and while they found no definitive mechanism for the colistin resistance in *A. baumannii*, the results for *Klebsiella* spp. revealed a modification of the *mgrB* gene by insertion sequence (IS): IS903B, inserted between nucleotide 107 and 108 for Kp65 and Kp82 isolates, and IS1R mobile element in the promoter region for Kp173, Kp174, Kp190, and Kp191 isolates [24]. In another, more recent study, 119 samples (oral, nasal, and rectal) were collected from 43 SARS-CoV-2 patients during the third wave of SARS-CoV-2 from May to June of 2021 at some SARS-CoV-2 isolation centers in the eastern part of Libya [37]. Using the Matrix-Assisted Laser Desorption/Ionization Time-of-Flight (MALDI-TOF) technique, *K. pneumoniae*, *Citrobacter Freundii*, *E. coli*, and *A. baumannii* constituted the prevalent bacterial isolates. Simene et al. screened these isolates for *mcr-1*, *mcr-2*, *mcr-3*, *mcr-4*, *mcr-5*, and *mcr-8* using PCR. The results showed that four out of seven *E. coli* isolates were colistin-resistant with an MIC of 4 mg/L, carrying the *mcr-1* gene. This marked the first reported *mcr-1* gene isolate in Libya. Colistin resistance mediated by a *mgrB* gene mutation was also noted in 3/32 *K. pneumoniae* isolates with MICs of between 16 mg/L and 32 mg/L using standard PCR and sequencing to amplify the entire *mgrB* gene [37].

### 3.5. Colistin Resistance in Iraq

There is not a great deal of data available on colistin resistance in Iraq. However, one research paper on multidrug resistance in military wounds occurring in Iraq and Afghanistan, published in 2008, discussed the emergence of colistin resistance, which had not previously been studied, because more than 95% of infected wound isolates were susceptible to colistin [38]. A literature review published in 2020 investigated the prevalence of colistin-resistant *K. pneumoniae* in the Middle East region and reported no instances of colistin resistance in Iraq [39].

### 3.6. Colistin Resistance in Oman

The first reported colistin-resistant *E. coli* strain (ST10) due to the *mcr-1* gene in the Sultanate of Oman was reported in a study conducted between July 2014 and June 2016. In this study, 21 *K. pneumoniae* isolates and one *E. coli* isolate were collected from a tertiary care hospital in Muscat, the capital of the country, and were screened for *mcr-1* and *mcr-2* genes using PCR. Among all the isolates, only one *E. coli* strain, isolated from the blood culture of a 58-year-old man who was treated for a nosocomial post-operative wound and bloodstream infections, was positive for the *mcr-1* gene [40]. A retrospective study was conducted between 1 January 2016 and 31 December 2017 at the Suhar Hospital to investigate bacterial resistance in northern Oman; 14 733 samples were collected, and 67.76% were Gram-negative (*E. coli*, *P. aeruginosa*, *K. pneumoniae*, and *A. baumannii*), 29% were Gram-positive, and 2.63% were *Candida* spp. Using the Kirby-Bauer disk diffusion technique, no resistance to colistin was shown in these isolates [41]. However, in another retrospective study on the prevalence and 30-day all-cause mortality of carbapenem- and colistin-resistant bacteremia caused by *A. baumannii*, *P. aeruginosa*, *and K. pneumoniae*, Balkhair et al. studied the resistance to both carbapenem and colistin of positive blood cultures for *A. baumannii*, *P. aeruginosa*, or *K. pneumoniae* in a 600-bed teaching and referral hospital in Oman. Among the 227 isolates that were found to be carbapenem-sensitive, 17 isolates (14:17 *K*. *pneumoniae* and 3:17 *P. aeruginosa*) were also resistant to colistin. The mechanism of resistance was not studied [42].

### 3.7. Colistin Resistance in Bahrain

In a study that focused on the Arab Peninsula, Sonnevand et al. investigated the presence of *mcr-1* genes in colistin-resistant *E. coli* isolates. The isolates were subjected to PCR for *mcr-1*; two isolates showed positive results, collected from a bed sore (August 2015) and urine (September 2015), respectively [43]. In a more recent study, Sahid et al. investigated the colistin resistance of carbapenem-resistant *K. pneumoniae (CRKP)* isolates. From tertiary care patients admitted to the Salmaniya Medical Complex in Bahrain between December 2020 and June 2021, 24 CRKP isolates were collected and subjected to MALDI-TOF and VITEK-2 tests for bacterial identification and antibiotic susceptibility, respectively. Sixteen of the 24 isolates exhibited resistance to colistin. For further investigation on the mechanism of resistance, Monoplex PCR (GeneAmp PCR System 9700) was conducted on these 16 colistin-resistant CRKP isolates to screen for *mcr-1*, *mcr-2*, and *mcr-3*. However, none of the isolates gave a positive result for any of these plasmid-mediated genes [44].

### 3.8. Colistin Resistance in Qatar

According to Forde et al., the first *mcr-1* positive *E. coli* strain in Qatar was isolated from a respiratory secretion sample from a 58-year-old man with subarachnoid hemorrhage admitted to the ICU in Qatar. The strain was identified as MS8345 or HZ-QTR-HMC-19. Using the automated broth microdilution technique (Vitek 2 card AST-N246), this strain was identified as being resistant to both beta-lactams and colistin [45]. Eighteen different samples of *K. pneumoniae* (*n* = 8), *E. coli* (*n* = 3), and *P. aeruginosa* (*n* = 2) were isolated from patients at the Hamad General Hospital in another study. Using the NMIC/ID-5 panel and the SensiTest colistin kit, the antimicrobial susceptibility of these isolates to colistin was determined and subsequently confirmed. Other techniques were used for whole genome sequencing (WGS) (BacPipe v1.2.6) and chromosomal modifications determination (CLC Genomics Workbench v.9.5.3). The results showed that two *K. pneumoniae* isolates possessed *mcr-8.1* on the IncFII(K) plasmids, three *K. pneumoniae* isolates had an insertion sequence (IS) element in the *mgrB* gene, one *E. coli* isolate possessed *mcr-1.1* on the IncI2 plasmid pEC12, and the other isolates showed chromosomal mutations in the *PhoPQ* TCS [46].

### 3.9. Colistin Resistance in the United Arab Emirates

In a study that focused on the Arab Peninsula, Sonnevend et al. studied the presence of the *mcr-1* gene in colistin-resistant *Enterobacteriaceae*. The PCR results showed that one *E. coli* (ABC149) isolate from a blood sample collected in May 2013 was resistant to colistin due to the presence of the plasmid-encoded *mcr-1* gene [43].

### 3.10. Colistin Resistance in Kuwait

There is not much data available on colistin resistance in Kuwait. The earliest data goes back to 2011 when 250 *Acinetobacter* isolates were collected from eight different governmental hospitals in Kuwait by Al-Suwaih et al. Using E-test, the susceptibility of these samples to colistin was determined. The results showed that 12% of these isolates were resistant to colistin [47]. Another study published in 2019 investigated the colistin resistance of 58 CRE-positive rectal swabs from patients admitted to the Adan and Mubarak Al Kabeer Hospitals in Kuwait between April 2017 and March 2018, within 48 h of their admission. Although the same susceptibility test was used as in previous studies, in this study, the resistance rate of colistin increased to 17% [48].

### 3.11. Colistin Resistance in Egypt

*mcr-1* was first reported in Egypt in 2016, where a sputum sample from a patient was shown to have an *E. coli* isolate with the *mcr-1* gene product [49]. A study conducted by Mai M. Zafer et al. at the National Cancer Institute at Cairo University, Egypt, between January 2016 and June 2017 sampled a total of 450 *K. pneumoniae* and *E. coli* strains from infected cancer patients. After isolation, the broth microdilution test showed that 40 isolates were colistin-resistant (8.8%). A genotypic survey of the 40 colistin-resistant isolates (18 *K. pneumoniae*, 22 *E. coli*) for the plasmid-encoded *mcr-1* and *mcr-2* genes revealed that only two showed the presence of the *mcr-1* gene. This *mcr-1* gene sequence was completely identical to the known *mcr-1* gene (GenBank: NG_050417.1). Genetic sequencing of *mgrB*, a regulator of the *PhoP/PhoQ* two-component system in 25 selected isolates, revealed the presence of a missense mutation *n* (GCC > GAC) replacing proline 178 with tyrosine in just one *K. pneumoniae* isolate. Two silent mutations were also present, TAA > CAA and a TCC > CCC, at positions 144 and 156, respectively [49].

Another study was conducted by Abozahra et al. where 82 *K. pneumoniae* isolates were collected from the Damanhour Medical National Institute and the Microbiology Department at the El Mery Hospital. Overall, 32 isolates showed colistin resistance as tested by broth microdilution. The presence of the *mcr-1* gene in the 32 colistin-resistant bacteria was searched for by PCR amplification, revealing that 27/32 isolates possessed the *mcr-1* gene. PCR assay and sequencing of the *mgrB* gene in 4/32 isolates not harboring the *mcr-1* gene were conducted. The assay showed that the four isolates had a premature stop codon that led to the premature termination of their translation during synthesis [50].

According to research conducted at the National Cancer Institute by Zafer et al., the frequency of colistin-resistant *K. pneumoniae* at the national level was 9.4% [49]. A study by Eriny T. Attalla et al. collected 17 colistin-resistant *Klebsiella* species (numbered K1-K17) which underwent WGS. The genetic modification of the *mgrB* locus was revealed to be the primary mechanism conferring colistin resistance, occurring in 82.35% of the collected isolates. The authors found that insertional inactivation by ISKpn14, a member of the IS1 family, was the predominant genetic alteration of the *mgrB* gene, while only two isolates had insertional inactivation by ISKpn26, a member of the IS5 family. Yet, the high prevalence of ISKpn14 contradicts previous research that found IS5 family components to be the primary source of *mgrB* inactivation. Eriny T. Attalla et al. found only one out of the 17 colistin-resistant isolates to possess the *mcr-1.1* gene [51].

### 3.12. Colistin Resistance in Saudi Arabia

Saudi Arabia is one of the biggest nations in the Middle East and welcomes millions of pilgrims every year for religious events including the Hajj [52]. Given the proposed correlation between hosting large religious gatherings and the increase in disease transmission [53,54], Alqasim et al. inferred that Saudi Arabia may be a focal point for the dissemination of drug-resistant bacteria worldwide. They also raised concerns about the scarcity of studies in Saudi Arabia addressing colistin resistance, despite its increasing global prevalence [53].

Recently, Okdah et al. characterized 52 colistin-resistant bacterial isolates from pulmonary samples from patients presenting at the King Fahad Medical Center. The cultured isolates were identified as *A. baumannii* (*n* = 37), *K. pneumoniae* (*n* = 10), and *P. aeruginosa* (*n* = 5). WGS of the *K. pneumoniae* isolates revealed several missense mutations in the *PhoP*/*PhoQ* regulator *mgrB*, *pmrA*, *pmrB*, and *phoQ* genes. However, in *A. baumannii* and *P. aeruginosa*, these mutations predominantly affected only one of the regulator genes, *pmrB*. Interestingly, the presence of the *mcr-1* gene was only noted in 20% of the *K. pneumoniae* isolates [55]. Similarly, Sonnevend et al. conducted pioneering work, discovering colistin resistance mediated by the *mcr-1* gene in extra-intestinal pathogenic *E. coli* (ExPEC) in Saudi Arabia [43]. Another study by Zaman et al. highlighted *mgrB* disturbances by insertional sequences, namely ISKpn14, ISKpn28, and IS903. Of the 23 colistin-resistant *K. pneumoniae* collected, 18 showed mutations in their *mgrB* gene while none harbored the *mcr-1* gene. Fifty-five percent of these mutations were caused by ISKpn14, 22% by ISKpn28 and 16% by IS903 [56].

### 3.13. Colistin Resistance in Jordan

A study in Jordan by Gharaibeh et al. tested for colistin resistance in *K. pneumoniae*. One hundred and seventy-nine clinical bacterial isolates, mainly collected from urine samples, were subjected to broth microdilution to test for their colistin susceptibility. Upon broth microdilution, 89 *K. pneumoniae* were identified as being resistant to colistin. PCR amplification for the *mcr-1*, *mcr-2*, *mcr-3*, *mcr-4*, *mcr-5*, *mcr-7*, and *mcr-8* genes was performed on the 89 *K. pneumoniae* isolates, revealing only one bacterium harboring an *mcr-1* gene. This may indicate that the majority of resistance to colistin is mediated by chromosomal genes rather than plasmid-mediated genes [57].

### 3.14. Colistin Resistance in Israel

In Israel, a study by Ben-Chetrit et al. investigated six colistin-resistant *K. pneumoniae* isolates. Upon WGS, none of the isolates were shown to possess any mobile colistin-resistant genes, but mutations in the *mgrB* gene were evident. Insertional sequence Kpn26 (ISKpn26) was identified in isolates 3 and 6 at the exact same position on their respective *mgrB* gene, thus disturbing the gene. WGS for isolates 4 and 5 revealed premature stop codons in the *mgrB* gene, but due to different mutations. In isolate 5, this was due to a single base-pair deletion at base 21, while in isolate 4 it was due to a single nucleotide polymorphism at base 48. Finally, isolates 1 and 2 had complete loss of *mgrB*, not only a disturbed protein [58].

At the Shaare Zedek Medical Center (Jerusalem, Israel), *15 K. pneumoniae* isolates were collected mostly through rectal swabs. Broth microdilution identified three isolates to be colistin-resistant with MIC ≥ 32 mg/L. Real-time PCR amplification for the *mcr-1* gene showed that none of the three isolates harbored it. After analysis of the *PhoP*/*PhoQ* regulator *mgrB* gene, two isolates had deleterious mutations, changing the amino acid sequence of the gene, while one had an IS5-like insertion inactivating the gene [59].

### 3.15. Colistin Resistance in Palestine

Between December 2018 and May 2019, 100 clinical isolates of *Enterobacteriaceae* were collected from different hospitals in the Gaza Strip. Broth microdilution was used to determine the colistin-resistant status of the isolates, where an MIC > 2 mg/L was classified as resistant. Broth microdilution identified 41 isolates (17 *E. coli*, two *Enterobacter*, 12 *Proteus*, four *Serratia*, and six *Klebsiella*) as being colistin-resistant [60].

### 3.16. Colistin Resistance in Lebanon

The first report of colistin-resistant *K. pneumoniae* in Lebanon was declared at the Sahel hospital in Beirut. Three clinical isolates, KP5, KP7, and KP106, were collected from cepacia medium (bioMérieux, Craponne, France), a medium selective for polymyxin-resistant isolates. Sequence analysis of the KP5 isolate revealed an insertion sequence (family ISEcp1) disturbing the associated colistin-resistant gene *mgrB*. However, for KP7, complete deletion of the *mgrB* gene was revealed in addition to a nucleotide insertion in *phoQ*, leading to a premature stop codon. The analysis of the KP106 isolate also showed a nonsynonymous mutation in the *pmrA* gene. Furthermore, a premature stop codon due to an amino acid deletion was identified in the *pmrB* gene of the KP106 isolate [61].

At the Saint George Hospital in Beirut, rectal swabs were taken from 23 patients being treated with a combination therapy of colistin and carbapenems. From the 23 rectal swabs, 12 isolates showed colistin-resistant enterobacteria, four of which were intrinsically resistant to colistin (one *Proteus mirabilis* and three *Morganella morganii*). Among these isolates, eight colistin-resistant strains were identified as five *E. coli*, two *E. cloacae*, and one strain of *K. pneumoniae*. PCR amplification of these eight strains showed no bacterial possession of the MCR genes (*mcr-1* to *mcr-5*). However, amplification and sequencing of colistin-resistance-associated genes (*mgrB*, *pmrA*, *pmrB*, *phoP*, and *phoQ*) showed nonsynonymous and deletion mutations among the eight isolates [62].

In Tripoli, 84 rectal swabs were collected from bakery workers in June 2018 (t0) to test for colistin-resistant *E. coli* (CO-E). Later, in December 2018 (t6), 70 out of the 84 bakery workers submitted another rectal swab. Susceptibility testing by the disk diffusion method on Mueller–Hinton agar at t0 showed that 5/84 rectal swabs were positive for colistin-resistant *E. coli* (CO-E), while at t6 only 1/84 came back positive for CO-E. No individual harbored colistin-resistant *E. coli* at the two time points simultaneously. PCR assays showed that the six CO-E positive swabs harbored the *mcr-1.1* gene [63]. Additionally, another study by Azour et al. investigated the presence of carbapenem- and colistin-resistant bacteria in Syrian refugees in Lebanon, revealing a concerning prevalence of resistance genes, including *bla*_OXA-48_, *bla*_NDM-1,_ and *mcr-1*, highlighting the urgent need to adopt strategies to prevent the spread of these MDR bacteria in overcrowded and underserved communities [6].

Colistin resistance in humans in the MENA region is summarized in Table 1.

## 4. Colistin Resistance in Animals in the MENA Region

**Figure 3:**
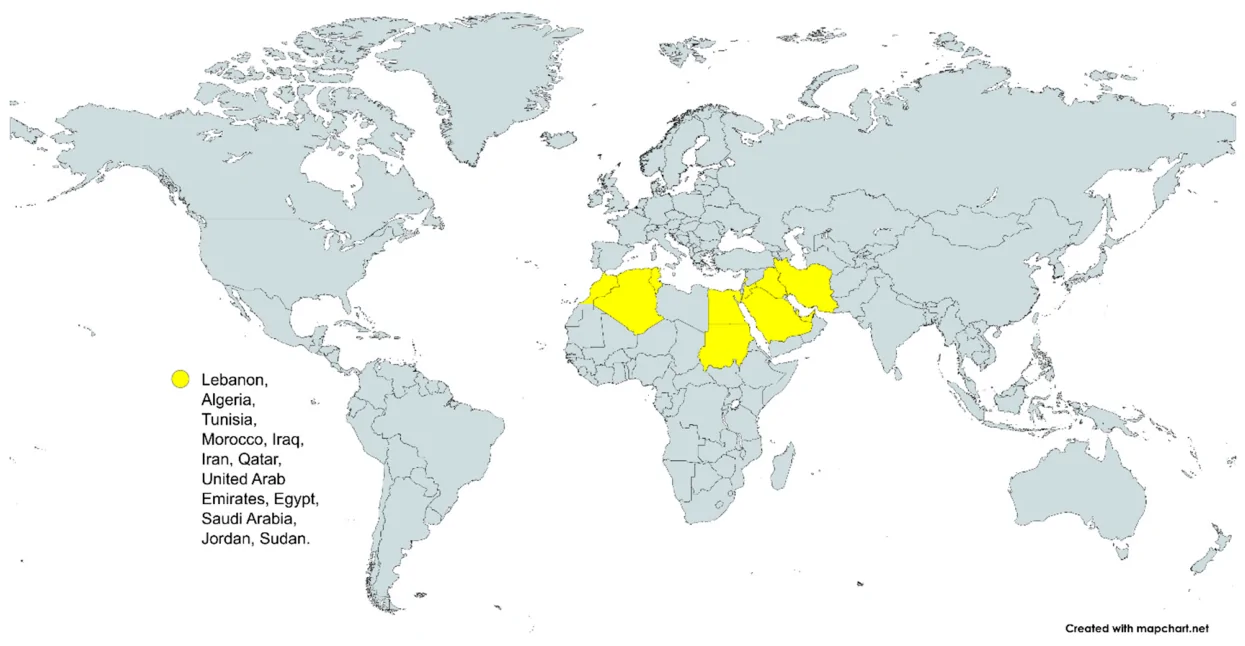
Global distribution of colistin resistance genes in animals: spotlight on the MENA region.

The extensive use of colistin in livestock, especially in poultry and aquaculture, has significantly contributed to the spread of resistance [64]. Studies have detected *mcr*-carrying bacteria in farms across the MENA region, posing a direct risk to public health, particularly through the food chain [4,65,66,67,68,69,70,71,72,73,74]. This section focuses on the available data regarding colistin resistance in farm animals and its connection to environmental factors (Figure 3).

### 4.1. Colistin Resistance in Animals in Tunisia

The presence of colistin resistance in GNB has been widely documented in Tunisia, and levels appear to be among the highest in northern Africa. This resistance has emerged in several animals, including chickens, bovines, cockroaches, camels, wild boars, and lastly farmed fish.

Colistin resistance was detected in both healthy broiler chicken samples (ST4187, ST3882, ST5693, and ST8932) and in chickens that had died from *colibacillosis/salmonella* spp. Further studies on these samples showed that 41.5% of the samples tested (81 out of 195) were colistin-resistant *E. coli* [65]. Furthermore, another study by Benlabidi et al. conducted on fecal swabs from free-range chickens reported two out of ten ESBL positive-*E. coli* isolates carrying the *mcr-2* gene. This study is the first to report the occurrence of *mcr-2* of animal origin in Tunisia [75]. Lastly, several studies have highlighted the presence of colistin resistance in chickens in Tunisia, albeit with different percentages of resistance, which are highly dependent on the sampling method.

Colistin resistance has also been detected in bovine fecal and milk samples. In a study conducted in 2019, it was found that four out of eight (three fecal samples ST1642 and one raw milk sample ST1642) ESBL positive-*E. coli* isolates (ST10) were colistin-resistant due to the presence of the *mcr-1* gene [76]. Another study, conducted on cows, showed that 36 out of 67 *E. coli* isolates were colistin-resistant, with bovine mastitis-associated isolates showing the greatest colistin resistance (18/25), and *mcr-1* gene was detected in only 10 isolates [77].

Moreover, in a study conducted by Landolsi et al., 144 cockroach samples from Tunisian hospitals were collected and studied. After isolation, nine *E. coli* samples (lineage 648) (6.25%) tested positive for colistin resistance due to the presence of the *mcr-1* gene on the IncHI2-type plasmid [78].

In another study conducted in 2018, 51 fecal samples were extracted from camel calves in southern Tunisia, and 28 (out of 51) were identified as possibly *E. coli* colistin-resistant. One sample of *E. cloacae* was confirmed to be resistant to colistin. The samples, however, showed neither the *mcr-1* nor the *mcr-2* genes [79]. In another study conducted in 2021 by Dziri et al., colistin-resistant *E. coli* isolates (ST162) were detected in camels on a farm in the south of the country. This resistance was conferred by the *mcr-1* gene on the IncHI2 plasmid [22]. Selmi et al. conducted a study on wild boar in Tunisia and showed that 3/102 enterobacterial isolates (*2 K. oxytoca* and *1 K. pneumoniae*) were resistant to colistin through the spread of the *mcr-1* and/or *mcr-2* genes [80].

Lastly, in their study, Zouiten et al. collected samples from aquaculture farms contaminated by colistin-resistant *Vibrio parahaemolyticus* and *Vibrio alginolyticus*. This occurrence is increasing due to the excessive use of colistin and other antibiotics in fish farms [81].

### 4.2. Colistin Resistance in Animals in Morocco

Baaboua et al. discussed the emergence of colistin resistance caused by either *Campylobacter jejuni* or *Campylobacter coli* appearing in various animals, including broiler chickens and turkeys. Firstly, it was found that 7.14% of *C. jejuni* broiler samples were resistant to colistin, as well as 2.82% of *C. coli* broiler samples, 4.17% of turkey samples, and 40% of various other samples [82]. Another study highlighted the presence of colistin resistance in fecal samples of broiler chickens by sampling 560 isolates, of which 26 were infected by *E. coli*, including three samples that appeared to have plasmid-mediated colistin resistance due to the presence of the *mcr-1* gene [66].

### 4.3. Colistin Resistance in Animals in Sudan

According to a single study by Feng et al. conducted on fennec foxes imported from Sudan to China for testing using broth microdilution, these animals are considered the biological vector for colistin resistance in the *Enterobacteriaceae* family. Testing on 88 fennec foxes imported to China revealed that 29 fecal samples were positive for *E. coli*, including a single colistin-resistant sample of the ST48 lineage (EcFF273) caused by the presence of the *mcr-1* gene on the plasmid IncI2 [67].

### 4.4. Colistin Resistance in Animals in Algeria

In Algeria, colistin resistance is highly prevalent due to the *mcr-1* gene and has been well documented in multiple animals, such as chickens, barbary macaques, and white storks. The *mcr-1* gene was first detected in chickens, and, according to Chabou et al. [68], it is important to highlight the presence of resistant *E. coli* isolates in Algerian poultry, which carry the plasmid-mediated colistin resistance gene *mcr-1*. Based on another study conducted by Chaalal et al., out of 181 samples of chicken meat collected in western Algeria, a total of 22 isolates (12.2%) tested positive for colistin resistance, including 17 *E. coli* (predominantly ST224) and five *K. pneumoniae* (ST17, ST646, and ST944). Moreover, the *mcr-1* gene was found in only 11 *E. coli* isolates and was associated with the IncFV and IncFIIK plasmids. In contrast, a single isolate showed high colistin resistance (MIC = 96 mg/L) with some new mutations in the chromosomal colistin-resistant genes [83]. Regarding barbary macaques, Bachiri et al. collected 86 fresh stool samples in the Toudja Forest, and a single *E. coli* M076 (ST405) isolate was found to be resistant to colistin by expressing the *mcr-1* gene. According to Bachhiri et al., this is the first description of the *mcr-1* gene from wildlife in Algeria and, indeed, Africa as a whole [84]. Lastly, Loucif et al. isolated and tested 42 *E. coli* isolates that were taken from white stork nests, and the results showed that 8/42 appeared to be colistin-resistant due to the presence of the *mcr-1* gene. However, eight distinct STs were identified by genotyping, including ST5542 (CC10), ST1286 (CC10), ST2973, ST58 (CC155), ST453 (CC86), ST9815, ST224, and the worldwide high-risk resistance lineage ST101 [85].

### 4.5. Colistin Resistance in Animals in Egypt

In a recent study by Ali et al., 250 samples of lungs and tracheas from chickens suffering from colisepticaemia, airsaccculitis, pericarditis, and perihepatitis were collected from 50 poultry farms situated in the eastern part of the Egyptian delta. Of the 250 diseased chickens, 140 samples (56%) tested positive for *E. coli*. After further antimicrobial susceptibility testing (AST), 115 samples exhibited colistin resistance, and 92 samples were ESBL-harboring *E. coli*. Additionally, 64 of the ESBL isolates (69.5%) were found to have the *mcr-1* gene. Genotyping and WGS revealed that serotype O125 was the most common, in addition to other serotypes such as O111, O86, and O157. The authors noted, however, that prior research in Egypt had documented different degrees of colistin resistance in *E. coli* bacteria in the northern governorates [4]. Another study on chickens in Egypt by El-Saeed et al. reported that 129 of the 356 investigated isolates were confirmed as *Salmonella* sp. Using PCR amplification, surprisingly, 82.2% (106/129) of these isolates were resistant to colistin [86]. Finally, Salem et al. tested the dissemination of the *mcr-1* gene *in P. aeruginosa* in both broiler chicks and dead-in-shell chick infections. This study resulted in the isolation of 55 *P. aeuriginosa* samples, and further testing confirmed that 18/55 samples (32.7%) were resistant to colistin, and 10/55 (18.2%) were found to express the *mcr-1* gene [87]. Demerdash et al. tested 317 birds from seven distinct districts in the Sharkia governorate (divided into 153 chickens, 36 quails, 38 turkeys, and 100 ducks). They were able to identify 30 *Pasteurella multocida* isolates (9.4%). Genotyping and WGS determined that these isolates were capsular type A, apart from one quail type D isolate. Most of these isolates exhibited high levels of resistance, reaching 60%, and the *mcr-1* gene was only detected in 12 of these samples (five chicken samples, four duck samples, two quail samples, and one turkey sample) [88]. Additionally, Ahmed et al. investigated the prevalence of colistin resistance among birds. This study included 80 resident wild birds divided equally into four groups of 20 (hooded crows, cattle egrets, rock pigeons, laughing doves), and 60 migratory waterfowl divided into three groups of 20 (shoveler ducks, Cotte ducks, and green-winged teal ducks), all collected from Giza, Cairo, and Ismailia and the ports of these governorates in Egypt. As a result of the sample extraction, isolation, and susceptibility testing, the *mcr* genes were detected in *E. coli*, *K. pneumoniae*, and *P. aeruginosa* from both resident and migratory birds. The prevalence of *mcr-1* in resident birds and migratory birds was 10.4% and 20%, while the prevalence of *mcr-2* was 1.4% and 3.6%, respectively [89]. Tartor et al. tested colistin resistance *mcr* genes in GNB isolated from bovine milk. They extracted milk samples from dairy cows showing mastitis, as well as from bulk-tank raw milk. Fifty-two percent of the 117 Gram-negative samples isolated (*Enterobacteriaceae n* = 90, *P. aeruginosa n* = 10, *A. hydrophila n* = 17) were colistin-resistant. This resistance was seen in 57.14% of *E. coli* samples, 77.78% *of K. pneumoniae* samples, 88.24% of *A. hydrophila* samples, and 80% of *P. aeruginosa* samples. After further testing including PCR and DNA sequencing, the *mcr-1* gene was identified in 31.9%, *mcr-2* in 29.8%, *mcr-3* in 34%, and both *mcr-4* and *mcr-7* in 2.13% of the resistant isolates [90]. According to another study, the same authors reported that *P. mirabilis* exhibited resistance in 12.2% of the samples (4/33 samples). Interestingly, this study represented the first isolation of an *mcr-10*-carrying *K. pneumoniae* isolate from a raw milk sample [91]. Sadek et al. discovered the presence of the *mcr-9* gene and its localization in *E. hormaechei* ST279, which was recovered from a beef patty in Egypt. This *mcr-9* gene, however, did not express any type of resistance to colistin, and the isolate was susceptible to colistin [92]. Another study by Sabeq et al. described the first report of colistin resistance phenotypes in *S. enterica* extracted from minced beef and rabbit meat. Besides detecting *mcr-1* in these samples, they also detected this gene in an egg [93]. Another study conducted in Cairo on 81 pig rectal swabs resulted in 47 ESBL-positive isolates (58%, 41 *E. coli* and six *K. pneumoniae*). After conducting AST, 11 *E. coli* isolates exhibited a colistin resistance phenotype, and all 11 isolates were *mcr-1* gene producers. This gene was localized on different plasmids, including IncI2, IncX4, and IncHI2 [94]. Finally, a study on camel meat in the Beheira governorate investigated 152 samples of meat taken from the hind quarter (topside region of the camel). The results showed that 45 of the 152 camel meat samples examined were infected with *Salmonella* sp. and 18.8% (nine samples) of these isolates exhibited resistance against polymyxin E [95]. Lastly, another study on camels conducted by Youseef et al. extracted and isolated 121 fecal swabs from camels in both the Cairo and Giza governorates. As a result, 75 *E. coli* isolates were identified. AST was conducted on these isolates and detected 29 colistin-resistant isolates. Genotyping and WGS revealed the prevalence of the *mcr* genes, with *mcr-1* being detected in three samples, *mcr-2* in two samples, *mcr-3* in 21 samples, and lastly *mcr-4* in three samples [96].

### 4.6. Colistin Resistance in Animals in Iraq

A single study in Iraq in 2020 by Kanaan et al. isolated 200 *A. baumannii* samples (turkey and chicken meat samples). After further testing, 12% of these samples presented colistin non-susceptibility due to the *mcr-1* gene. They added that two colistin-susceptible *A. baumannii* isolates were found to be carrying the *mcr-1* gene [69].

### 4.7. Colistin Resistance in Animals in Lebanon

The level of resistance detected among the Lebanese poultry sector might be one of the highest in the region. One study conducted on broiler farms by Hmede et al. extracted 93 samples, of which 97% (90 samples) yielded *E. coli* colonies. Isolation of these colonies showed that 88 isolates harbored the *mcr-1* gene, indicating widespread resistance to colistin [70]. Another study conducted multiple tests on fresh chicken wings in Lebanon, including susceptibility and genome sequencing, after collecting 180 samples of fresh chicken meat from 52 retail stores and 183 fecal matter samples from six broiler farms. Isolation of the samples led to the identification of *E. coli* ST58 (ST3107), and further susceptibility testing revealed colistin resistance due to the *mcr-1* and *mcr-1.26* genes. Furthermore, WGS detected that *mcr-1.26* is transmissible and is located on an IncX4 plasmid. According to Kassem et al., this might be the first report of *mcr-1.26* in poultry meat worldwide. The authors added that *mcr-1.26* has previously been reported in bacteria isolated from the fecal droppings of a domesticated pigeon in Lebanon, in addition to other clinical samples, suggesting that it might be spreading to different hosts [97]. Another study conducted in south Lebanon by Dandachi et al. on swine farms investigated 94 fecal samples from pigs; *E. coli* was detected in 22 samples, in addition to *K. pneumoniae* in one sample, and all were resistant to colistin and carried the *mcr-1* gene on the plasmids [98]. Lastly, a publication by Hassan et al. reported the presence of the *mcr-1.1* gene in farmed rainbow trout. However, the number of fish tested was limited, which restricts generalizing the findings to all farms in Lebanon. The *mcr-1.1* gene was present on the transmissible IncX4 plasmids in five MDR *E. coli* isolates [99].

### 4.8. Colistin Resistance in Animals in Qatar

According to Eltai et al., and based on a study conducted on cloacal chicken samples collected from three different farms in Qatar, *E. coli* was detected in 90 samples out of 172 (52%). After further isolation and susceptibility testing, 14 out of the 90 samples (15.6%) were found to be resistant to colistin, mediated by the *mcr-1* gene. The *mcr-2* gene was absent from all of the isolates [71]. Another study by Eltai et al. conducted in Qatar on imported retail chicken carcasses also detected the presence of colistin resistance mediated by the *mcr-1* gene on the plasmids of 69 resistant samples out of 216 (31.9%). The prevalence of this resistance appears to be increasing with time, which is very worrying, since it is considered a last-resort antibiotic. This study also compared the prevalence between both locally chilled chicken *E. coli* isolates and imported chilled and frozen chicken. The locally chilled *E. coli* isolates presented the highest prevalence of resistance, at 81.2%, compared to 13% among imported chilled isolates and 5.8% among imported frozen isolates [100]. Finally, in a study aiming to search for antimicrobial resistance in commensal *E. coli* isolated from food animals in Qatar, AlHalabi et al. discovered a single *E. coli* isolate from a pigeon that was resistant to colistin due to the presence of the *mcr-1* gene [101].

### 4.9. Colistin Resistance in Animals in Jordan

In Jordan, a study conducted by Gharaibeh et al. on the presence of *mcr* genes in the animal sector was the first to prove the presence of *mcr-1* in *E. coli* in the poultry industry in Jordan. This study extracted cloacal samples from 385 chickens in 125 poultry farms, and of these samples, 360 were positive for *E. coli*, of which 58% (209 samples) were colistin-resistant, either presenting the *mcr-1* gene (83 = 39.7% were *mcr-1*, which was the only type detected) or were found to be lacking this gene (126 samples, 60.3%) [72]. Another study by Obaidat et al. reported that Jordan imports its fish mainly from three countries: Yemen, India, and Egypt. After testing imported and freshly caught fish samples, *V. parahaemolyticus*, which is a leading cause of seafood-associated illness, was detected in 14.9% of the 330 samples, with the highest prevalence coming from Yemen. Obaidat et al. added that all isolates were resistant to colistin [102].

### 4.10. Colistin Resistance in Animals in Saudi Arabia

A study by Yasir et al. collected 40 poultry meat samples from a local market in Jeddah, Saudi Arabia. Further research was conducted, which confirmed the presence of colistin resistance due to the presence of the *mcr-1.1* gene in the *E. coli* isolate Ch7_4SAIU, which is classified into ST3270 [103].

### 4.11. Colistin Resistance in Animals in the United Arab Emirates

A study by Habib et al. isolated 17 ESBL-producing *E. coli* strains from healthy pets in the UAE, which were investigated using WGS. They subsequently detected plasmids harboring the mobilized gene *mcr-1.1* conferring *E. coli* resistance against colistin in these samples. The presence of this gene was confirmed in two isolates out of 17, which belonged to ST1011 within phylogroup D. This report was the first to detect *mcr1.1* in pets in both the UAE and the Middle East more widely [104]. Another study conducted in the UAE by Habib et al. included 315 chicken samples. These samples were extracted from seven different brands of chicken, six of which are established in the UAE and one which imports its chicken from Saudi Arabia. The results showed that 7% (7/100) of the isolates were resistant to colistin due to the presence of the *mcr-1* gene [105]. Another study conducted on fecal samples from healthy broiler chickens in Abu Dhabi by Sonnevand et al. reported the collection of 219 colistin-resistant samples. They then selected 39 samples and conducted further studies confirming the presence of the *mcr-1.1* gene. These samples included 34 *E. coli*, two *Escherichia albertii*, two *K. pneumoniae* (ST340), and one *S. enterica* (Serovar Minnesota). This *mcr-1.1* gene was found to be prevalent on different plasmids such as incI2, IncHI2, and IncX4 [73]. Other studies also mentioned the presence of two *mcr-1.1* genes in *S. enterica* (Serovar Minnesota) isolated from retail chicken meat [73].

### 4.12. Colistin Resistance in Animals in Iran

In Iran, Nikkhahi et al. studied the prevalence of the *mcr-1* gene in *E. coli* in livestock. A total of 115 samples were extracted, including 38 cow samples and 47 chicken samples. Subsequently, AST showed that only one cow isolate and one chicken isolate were resistant to colistin. PCR was performed on these isolates and revealed that only one isolate from cows harbored the *mcr-1* gene. No other *mcr* genes were detected [74]. Another study conducted in Iran by Dadar et al. reported that colistin resistance was present in 100% of the samples extracted: 40 samples divided into 23 human samples and 17 animal isolates (*n* = 17) obtained from milk samples (6 cows, 1 camel, and 1 sheep), lymph nodes (3 cows and 1 camel), and aborted fetuses (4 sheep and 1 goat) [106]. Further studies, including PCR and WGS, identified that these samples were infected with *Brucella melitensis* [106]. In another study, 944 samples were collected from chicken farms located in the East Azerbaijan province in Iran, including 504 samples from healthy broilers at slaughterhouses and 441 samples from dead poultry referred to clinics, totaling 931 isolates of enteric bacterial samples that were screened for colistin resistance. After further biochemical testing, they identified nine colistin-resistant bacteria (0.96%), all identified as *K. pneumoniae*. After further investigation, the nine samples were shown to belong to seven different strains, with the main domination of ST11 (22.2%) [107]. Colistin resistance in *Salmonella enteritidis* was also detected in 15 out of 63 (23.8%) poultry and egg samples. Moreover, resistance was also detected in 23 (100%) of poultry samples contaminated by *Salmonella infantis.* Although colistin resistance was prevalent in both *Salmonella* and *Klebsiella*, the *mcr-1* and *mcr-2* genes were never detected [108]. Kanaan et al. detected resistance caused *by A. baumannii* among 12% of the 200 samples isolated, including 120 turkey samples and 80 chicken samples, partly conferred by the presence of the *mcr-1* gene [69].

Colistin resistance in animals in the MENA region is summarized in Table 2.

## 5. Colistin Resistance in the MENA Region in Water, Soil, and the Environment

Bacterial colistin resistance has become a significant public health concern worldwide, including in the MENA region. This resistance is of growing concern, since human health is closely connected to the health of animals and our shared environment.

Various water habitats, including wastewater, surface water, and tap water, could be a reservoir and route for the dissemination of mobile colistin-resistant genes (*mcr*). An overview of the global epidemiology of *mcr* genes in these environments was provided by Cherak et al., emphasizing the need for a better understanding of the role of the water environment in the spread of antibiotic resistance [109].

**Figure 4:**
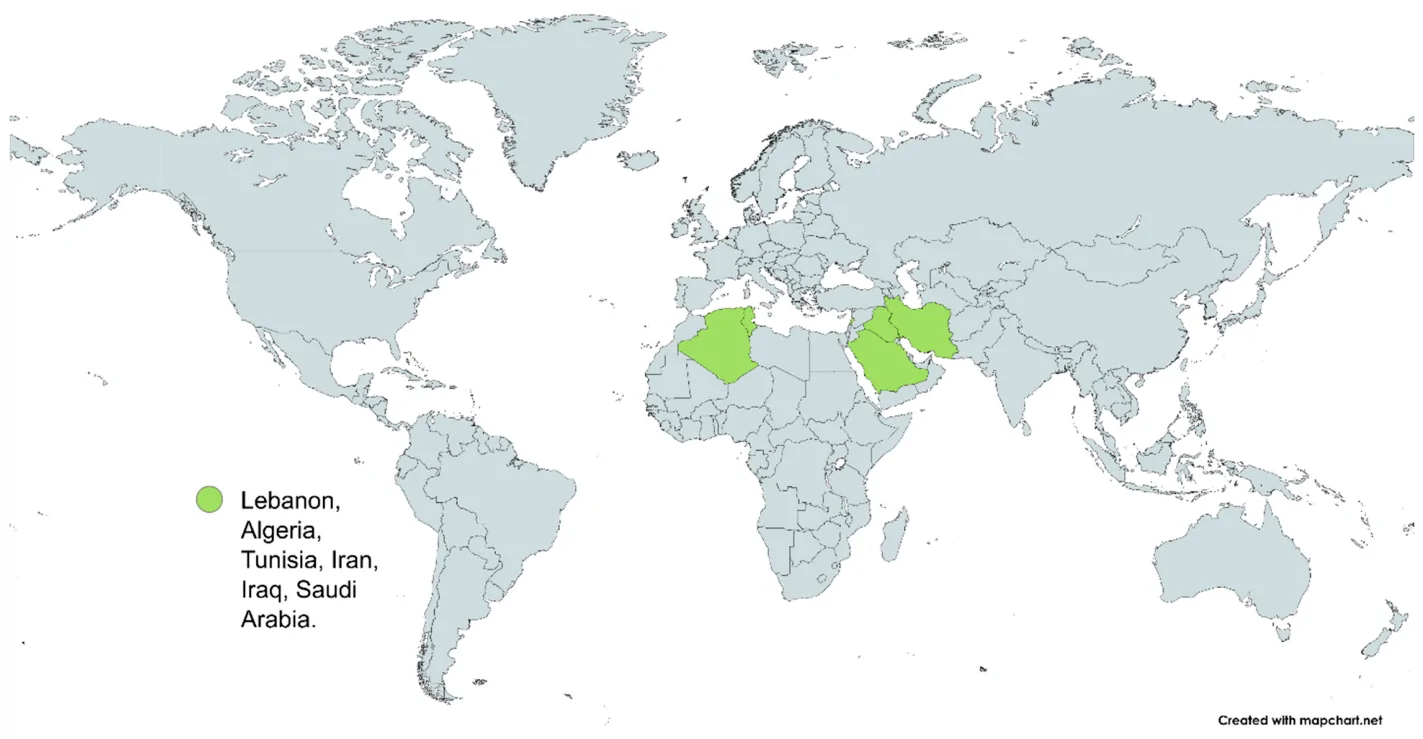
Global distribution of colistin resistance genes in water, soil and the environment: spotlight on the MENA region.

Bacteria have obtained *mcr* genes (*mcr-1* to *mcr-9*), transferring them into environments such as water bodies, soil, plants, wildlife habitats, animal environments, and public areas. According to Anyawu et al., [110], these genes have been found in several bacterial species, including *E. coli*, *Enterobacter*, *Klebsiella*, *Proteus*, *Salmonella*, *Citrobacter*, *Pseudomonas*, *Acinetobacter*, *Kluyvera*, *Aeromonas*, *Providencia*, and *Raulotella* isolates [110] (Figure 4).

### 5.1. Colistin Resistance in the Environment in Algeria

In the data collected about colistin resistance in the environment in Algeria, [3] demonstrating *mcr* gene resistance, Drali et al. reported a first case of *mcr-1*-carrying *E. coli* ST23 and ST115 from seawater sampled from the Algerian coast, during the summer of 2016. The samples, taken from 62 beaches along the Algiers coast, highlighted the global spread of the gene and the need for improved wastewater and sewage treatment protocols [3,111].

During September and October 2019, another study was conducted by Cherak et al., who reported the first detection of an *mcr*-5 gene in an unusual bacterial isolate, *Cupriavidus gilardii*, recovered from well water supplying a hospital located in an urban region in Batna, Algeria. This study sheds light on this significant environmental reservoir of *mcr* genes [112].

### 5.2. Colistin Resistance in the Environment in Iran

Although there is limited data available about colistin resistance in water and the environment in Iran, a cohort study aimed to investigate the prevalence of antibiotic resistance in *E. coli* strains isolated from humans, animals, food, and the environment. The results showed that environmental isolates had the lowest percentage of colistin resistance [113].

### 5.3. Colistin Resistance in the Environment in Iraq

A first report in Iraq by Al-Kadmy et al. aimed to identify genes associated with colistin resistance in *A. baumannii*, analyzing a few environmental isolates in addition to clinical isolates in Baghdad between 2016 and 2018. Their results showed a high prevalence (76%) of colistin resistance. Molecular analysis revealed the presence of the *mcr-1*, *mcr-2*, and *mcr-3* genes in a significant proportion of the isolates, indicating a genetic basis for resistance. The findings underscore the urgency of prudent colistin use to mitigate the spread of resistant strains [114].

### 5.4. Colistin Resistance in the Environment in Kuwait

One study focused on the isolation of antibiotic-resistant bacteria from the atmospheric air in hospital wards and outdoor areas in Kuwait during sandstorms in 2021. This study, by Shamsah et al., isolated eighty-four colonies, including *Staphylococcus aureus*, *coagulase-negative Staphylococcus*, *Bacillus* spp., and *Acinetobacter* spp. from hospital air, and *Pseudomonas* spp. and *Staphylococcus* spp. from outdoor air. The results revealed that none of the isolates showed colistin resistance [115].

### 5.5. Colistin Resistance in the Environment in Saudi Arabia

In a study aiming to screen for antibiotic resistance genes in bacteria in the upstream and downstream areas of the Wadi Hanifah valley in Riyadh, Saudi Arabia, Al-Otaibi et al. searched for the presence of the *mcr-1* gene in that area. Water samples were obtained from 18 distinct sites in Wadi Hanifah in September 2022. Following DNA extraction, the *mcr-1* gene was detected but at very low prevalence [116].

### 5.6. Colistin Resistance in the Environment in Tunisia

A study conducted by Hassen et al. in Tunisia reported the *mcr-1* gene in ESBL-producing *E. coli* from wastewater for the first time [5]. Between 2017 and 2019, samples were collected at different points along the sewage treatment process from the municipal wastewater treatment plant of La Charguia, which receives wastewater of various types such as domestic, hospital, urban, rain, and industrial waters from various areas of the city of greater Tunis. The results revealed that seven isolates were colistin-resistant (three *E. coli*, three *K. pneumoniae*, and one *E. cloacae* complex). Among these isolates, only one *E. coli* harbored the *mcr-1* gene (COL MIC = 16 μg/mL), recovered from raw water. The ISApl1 genetic element and the *pap2* gene were located in the upstream and the downstream regions of the *mcr-1* gene, respectively. The remaining six colistin-resistant isolates were negative for the *mcr-1* to *mcr-8* genes. The occurrence and prevalence of the *mcr*-1 gene in wastewater treatment plants and in the natural environment are still largely unknown [5].

### 5.7. Colistin Resistance in the Environment in Lebanon

Several studies from Lebanon have revealed the detection of the plasmid-mediated colistin resistance gene *mcr*-1 and *mcr*-*1.1* in *E. coli* isolates collected from irrigation water and from domestic and sewer waters in Syrian refugee camps in Lebanon [117,118,119,120].

Other studies have shown the detection of the plasmid-borne colistin resistance gene *mcr*-1 in *P. mirabilis* isolated from both domestic and sewage waters in Syrian refugee camps [121].

In the first study [117], irrigation water samples were collected from two major agricultural areas in Lebanon, namely south Lebanon and the Beqaa Valley between April and September 2018. The results were positive for the *mcr*-1 gene, which was further confirmed by sequencing, although the genes *mcr-2*, *-3*, *-4*, *-5*, *-6*, *-7* and *-8* were not detected in any isolate. The prevalence of *mcr-1*-positive *E. coli* detected in Lebanese irrigation water is higher in comparison with other countries. The draft genome sequences are important for understanding the dissemination of colistin-resistant *E. coli* strains and plasmid-borne *mcr-1.1* in agricultural waters, which might select for antibiotic resistance determinants that persist in the food chain [119]. This highlights the need to enhance and implement efficient antimicrobial stewardship and surveillance programs to control the proliferation of resistance to colistin and other critical antibiotics via agricultural practices and environmental matrices, since Lebanese irrigation water might affect a variety of environments, including the Mediterranean Basin, and the widespread occurrence of *mcr-1* might be a cause for concern.

Composite samples (1 L) of drinking-, well-, and camp-generated sewer waters were collected from two Syrian camps in Lebanon. Results showed a probable cross-contamination that may have occurred between the different sources (drinking water, well water, and sewage). The selected *mcr-1*-positive isolates survived in autoclaved drinking- and well-waters for more than 30 days without losing *mcr-1* and colistin resistance, suggesting that *mcr-1* might persist in these waters. Two MDR *E. coli* strains with the plasmid-borne colistin resistance gene (*mcr-1.1*) were isolated from the waters used by refugees and sequenced to analyze antibiotic resistance determinants. It was shown that the *E. coli* strains carried 15 and 20 AMR genes, including *mcr-1.1* and others that encoded resistance to important classes of antibiotics. Two MDR *E. coli* strains carrying the *mcr-1.1* gene were isolated from water used by refugees, highlighting the urgent need for these populations to have access to clean water [118,120].

Another report highlighted the presence of the plasmid-borne colistin resistance gene (*mcr-1*) in *P. mirabilis* isolated from domestic and sewer waters in Syrian refugee camps [15]. Since *P. mirabilis* is essentially colistin-resistant and not normally screened for *mcr-1*, the bacterium can act as an unnoticed reservoir for the transmission of *mcr-1* to colistin-susceptible bacteria in the camps and beyond (through sewer effluents and cross-contamination).

Contaminated domestic water raises the risk of disease and complicates the treatment of infections among vulnerable populations. Polluted sewage systems may contribute to the spread of MDR strains beyond refugee camps. The rising resistance to colistin and other critical antibiotics presents serious risks to both Syrian refugees and host communities. As a result, immediate action is required to secure sustainable access to clean water and ensure the effective management of wastewater [118,120].

Previous studies have reported that the wide dissemination of *mcr-1* across Lebanon is a major concern, especially in agricultural areas. Recently, a new *mcr* variant, *mcr-1.26*, was detected, which appears to be exclusive to Lebanon and has been rarely reported abroad. This gene-harboring bacterium appeared to simultaneously exhibit resistance to other clinically and agriculturally important antimicrobials [122].

Although data on the *mcr* gene in aquaculture are limited, the detection of the MCR gene *mcr-1.1* on IncX4 plasmids in MDR *E. coli* isolated from farmed rainbow trout showed that there is a high potential for the evolution and dissemination of *mcr* via aquatic environments, leading to a pressing need to control *mcr* and enhance antimicrobial stewardship in Lebanon [99].

Colistin resistance in water, soil, and the environment in the MENA region is summarized in Table 3.

## 6. Conclusions

In conclusion, colistin resistance has emerged as a critical public health challenge in the MENA region. Once considered a last-resort antibiotic for treating MDR GNB, the efficacy of colistin has been increasingly compromised by the rise in resistance, driven by its widespread use in agriculture, veterinary practices, and human healthcare. The dissemination of mcr genes, particularly the *mcr* gene family, has exacerbated the situation by enabling the rapid transfer of resistance traits across bacterial species, further limiting therapeutic options.

In the MENA region, countries such as Tunisia, Algeria, and Egypt are facing an alarmingly high rate of colistin-resistant bacteria, particularly within hospital settings where infections caused by *E. coli*, *K. pneumoniae*, and *A. baumannii* are prevalent. The prevalence of resistance in livestock and agricultural environments highlights the significant role of animal farming in the spread of resistant strains. The presence of *mcr* genes in animals and food products, particularly poultry, underscores the risk of transmission along the food chain, posing a direct threat to public health.

Environmental factors also contribute to the propagation of colistin resistance. Studies have shown that untreated wastewater, irrigation systems, and sewage can serve as reservoirs for resistant bacteria, facilitating their transfer between humans, animals, and the environment. The “One Health” approach emphasizes the need for a unified strategy to address antimicrobial resistance, where interventions target not only hospital settings but also agricultural practices and environmental contamination.

The current situation demands urgent action. It is crucial to strengthen antimicrobial stewardship programs to prevent the misuse of colistin in both healthcare and agriculture. Surveillance systems need to be reinforced to track resistance trends across the human, animal, and environmental interfaces, providing real-time data to guide policy decisions. Investments in research for alternative therapeutic options, such as new antibiotics, combination therapies, and phage therapy, must be prioritized, as the effectiveness of existing drugs continues to diminish.

Ultimately, addressing colistin resistance in the MENA region requires a collaborative effort from policymakers, healthcare professionals, veterinarians, and the agricultural sector. By adopting a comprehensive and coordinated strategy, it may be possible to slow the spread of resistance and preserve the efficacy of one of the last remaining antibiotics effective against MDR infections.

## Figures and Tables

**Table 1:** Overview of bacterial species and colistin resistance mechanisms in human isolates by country.

Country	Species	Resistance Mechanisms	Report Date	Reference
Algeria	*E. coli*	*mcr-1* gene	2011	[25]
	*A. baumannii*	*pmrB* mutation	2014	[26]
Tunisia	*Enterobacteriaceae*	*mcr-1* gene	2023	[29]
	*Enterobacteriaceae*	*mgrB* mutation (insertion)	2016	[30]
	*K. pneumoniae*	N/A	2017	[31]
	*K. pneumoniae*	*mcr-1* gene	2018	[32]
	*A. baumannii*	*pmrA* and *pmrB* mutation	2018	[33]
	Gram-bacilli	*mcr-1* gene	2022	[34]
Morocco	*E. coli*	*mcr-1* gene	2014	[35]
Libya	*A. baumannii* and *Klebsiella* spp.	N/A	2018	[36]
	*Klebsiella* spp.	*mgrB* mutation (insertion)	2023	[24]
	Gram-bacilli	*mcr-1* gene	2023	[37]
Iraq	*K. pneumoniae*	*mcr-1* gene	2023	[39]
Bahrain	*K. pneumoniae*	No resistance	2022	[44]
	*E. coli*	*mcr-1* gene	2016	[43]
Qatar	*K. pneumoniae*	N/A	2023	[46]
	*E. coli*	*mcr-1* gene	2018	[45]
UAE	*E. coli*	*mcr-1* gene	2016	[43]
Kuwait	*Acinetobacter*	N/A	2011	[47]
	colistin-resistant *Enterobacteriaceae*	N/A	2020	[48]
Egypt	*E. coli* and *K. pneumoniae*	*mcr-1* gene and *mgrB* missense mutation	2019	[49]
	*K. pneumoniae*	*mcr-1* gene	2023	[50]
	*K. pneumoniae*	*mcr-1.1* gene and *mgrB* mutation	2023	[51]
KSA	*E. coli*	*mcr-1* gene	2016	[43]
	Gram-bacteria	*mcr* gene and *pmrA*, *pmrB*, and *phop/Q* genes mutation	2021	[53]
	Gram-bacteria	*mcr* gene and *pmrA*, *pmrB*, and *phop/Q* genes mutation	2014	[54]
	*Klebsiella*, *Acinetobacter* and *Pseudomonas*	*pmrA*, *pmrB*, and *phop/Q* genes mutation	2022	[55]
	*Klebsiella*	*mcr* gene	2018	[56]
Jordan	*K. pneumoniae*	*mcr-1* gene	2022	[57]
Israel	*K. pneumoniae*	*mgrB* mutation	2021	[58]
	*K. pneumoniae*	*mgrB* mutation	2019	[59]
Palestine	*K. pneumoniae*	*mgrB* mutation	2021	[60]
Lebanon	*k. pneumoniae*	*mgrB*, *pmrA*, and *pmrB* gene mutations	2017	[61]
	*Enterobacteriaceae*	*mgrB*, *pmrA*, *pmrB*, and *phoQ* genes mutation	2020	[62]
	*E. coli*	*mcr-1.1* gene	2021	[63]

UAE: United Arab Emirates; KSA: Kingdom of Saudi Arabia; *E. coli*: *Escherichia coli*; *A. baumannii*: *Acinetobacter baumannii*; *K. pneumoniae*: *Klebsiella pneumoniae*; *P. aeruginosa*: *Pseudomonas aeruginosa*; *P. mirabilis*: *Proteus mirabilis*; *E. cloacae*: *Enterobacter cloacae*; *M. morganii*: *Morganella morganii*; *C. freundii*: *Citrobacter freundii*; N/A: Not applicable; mcr: mobile colistin resistance; pmrA/B: polymyxin resistance regulator A/polymyxin resistance sensor kinase B; PhoP/PhoQ: PhoP–PhoQ two-component regulatory system; mgrB: PhoP–PhoQ system regulator MgrB.

**Table 2:** Overview of bacterial species and colistin resistance mechanisms in animal isolates by country.

Country	Animal	Species	Resistance Mechanism	Reporting Date	Reference
Tunisia	Chicken	*E. coli*	N/A	2023	[65]
	Chicken	ESBL *E. coli*	*mcr-2* gene	2023	[75]
	Bovine	ESBL *E. coli*	*mcr-1* gene	2019	[76]
	Cow	*E. coli*	*mcr-1* gene	2023	[77]
	Cockroach	*E. coli*	*mcr-1* gene	2022	[78]
	Camel calf	*E. cloacae*	N/A	2018	[79]
	Camel calf	*E. coli*	*mcr-1* gene	2021	[22]
	Wild boar	*K. oxytoca*	*mcr-1* and/or *mcr-2* gene	2022	[80]
	Wild boar	*K. pneumoniae*	*mcr-1* and/or *mcr-2* gene	2022	[86]
	Farm fish	*V. parahaemolyticus*	N/A	2017	[81]
	Farm fish	*V. alginolyticus*	N/A	2017	[81]
Morocco	Chicken	*E. coli*	*mcr-1* gene	2018	[66]
	Chicken	*C. jejuni*	N/A	2021	[82]
	Chicken	*C. coli*	N/A	2021	[82]
	Turkey	*C. coli*	N/A	2021	[82]
Sudan	Fox	*E. coli*	*mcr-1* gene	2019	[67]
Algeria	Chicken	*E. coli*	*mcr-1* gene	2021	[68]
	Chicken	*K. pneumoniae*	N/A	2021	[83]
	Barbary macaques	*E. coli*	*mcr-1* gene	2018	[84]
	White stork	*E. coli*	*mcr-1* gene	2022	[85]
Egypt	Chicken	*E. coli*	*mcr-1* gene	2024	[4]
	Chicken	*Salmonella*	N/A	2024	[86]
	Chicken	*P. aeruginosa*	*mcr-1* gene	2024	[87]
	Birds	*P. multicoda*	*mcr-1* gene	2023	[88]
	Birds	*E. coli/K. pneumoniae/P. aeruginosa*	*mcr-1* gene	2019	[89]
	Birds	*E. coli/K. pneumoniae/P. aeruginosa*	*mcr-2* gene	2019	[89]
	Bovine	*E. coli*	*mcr-1/2/3/4/7* genes	2021	[90]
	Bovine	*K. pneumoniae*	*mcr-1/2/3/4/7* genes	2021	[90]
	Bovine	*A. hydrophila*	*mcr-1/2/3/4/7* genes	2021	[90]
	Bovine	*P. aeruginosa*	*mcr-1/2/3/4/7* genes	2021	[90]
	Bovine	*P. mirabilis*	*mcr-10* gene	2021	[91]
	Cattle/Rabbit	*Salmonella*	*mcr-1* gene	2022	[93]
	Pig	*E. coli*	*mcr-1* gene	2022	[94]
	Camel	*Salmonella*	N/A	2022	[95]
	Camel	*E. coli*	*mcr-1/2/3/4* genes	2024	[96]
Iraq	Chicken	*A. baumannii*	*mcr-1* gene	2020	[69]
	Turkey	*A. baumannii*	*mcr-1* gene	2020	[69]
Lebanon	Chicken	*E. coli*	*mcr-1* gene	2018	[70]
	Chicken	*E. coli*	*mcr-1* gene	2023	[97]
	Swine	*E. coli*	*mcr-1* gene	2018	[98]
	Swine	*K. pneumoniae*	*mcr-1* gene	2018	[98]
	Rainbow trout	MDR *E. coli*	*mcr-1* gene	2020	[99]
Qatar	Chicken	*E. coli*	*mcr-1* gene	2018	[71]
	Retail chicken	*E. coli*	*mcr-1* gene	2020	[100]
	Pigeon	*E. coli*	*mcr-1* gene	2020	[101]
Jordan	Chicken	*E. coli*	*mcr-1* gene	2024	[72]
	Fish	*V. parahaemolyticus*	N/A	2017	[102]
KSA	Chicken	*E. coli*	*mcr-1.1* gene	2021	[103]
UAE	Pets	*E. coli*	*mcr-1.1* gene	2023	[104]
	Chicken	N/A	*mcr-1* gene	2023	[105]
	Chicken	*S. enterica*	*mcr-1.1* gene	2022	[73]
	Chicken	*E. coli*	*mcr-1.1* gene	2022	[73]
	Chicken	*E. albertii*	*mcr-1.1* gene	2022	[73]
	Chicken	*K. pneumoniae*	*mcr-1.1* gene	2022	[73]
	Chicken	*S. enterica*	*mcr-1.1* gene	2022	[73]
Iran	Cow	*E. coli*	*mcr-1* gene	2021	[74]
	Chicken	*E. coli*	N/A	2021	[74]
	Bovine	*B. melitensis*	N/A	2023	[106]
	Camel	*B. melitensis*	N/A	2023	[106]
	Chicken	*K. pneumoniae*	N/A	2019	[107]
	Chicken	*S. infantis*	N/A	2021	[108]
	Chicken	*S. enteridis*	N/A	2021	[108]
	Turkey	*A. baumannii*	*mcr-1* gene	2020	[69]
	Chicken	*A. baumannii*	*mcr-1* gene	2020	[69]

ESBL: Extended-Spectrum Beta-Lactamase; N/A: not applicable; MDR: Multi-Drug Resistant; UAE: United Arab Emirates; *E. coli*: *Escherichia coli*; *E. cloacae*: *Enterobacter cloacae*; *K. oxytoca*: *Klebsiella oxytoca*; *K. pneumoniae*: *Klebsiella pneumoniae.*; *V. parahaemolyticus*: *Vibrio parahaemolyticus*; *V. alginolyticus*: *Vibrio alginolyticus*; *C. jejuni*: *Campylobacter jejuni*; *C. coli*: *Campylobacter coli*; *P. aeruginosa*: *Pseudomonas aeruginosa*; KSA: Kingdom of Saudi Arabia; *P. multicoda*: *Pasteurella multicoda*; *A. hydrophila*: *Aeromonas hydrophila*; *P. mirabilis*: *Proteus mirabilis*; *A. baumannii*: *Acinetobacter baumannii*; *S. enterica*: *Salmonella enterica*; *E. albertii*: *Escherichia albertii*; *B. melitensis*: *Brucella melitensis*; *S. infantis*: *Salmonella infantis*; *S. enteridis*: *Salmonella enteridis*.

**Table 3:** Overview of bacterial species and colistin resistance mechanisms in water, soil and environmental isolates by country.

Country	Source	Species	Resistance Mechanism	Report Date	Reference
Algeria	Sea water	*E. coli* ST23 and ST115	*mcr-1* gene	2016	[3,111]
	Well water supplied to hospital	*Cupriavidus gilardii*	*mcr-5.1* gene	2019	[112]
Iran	Water in environment	MDR-ESBL *E. coli*	—	2019	[113]
Iraq	Environment	*A. baumannii*	*mcr-1*, *mcr-2*, *mcr-3* genes	2020	[114]
Kuwait	Hospital air	*Staphylococcus aureus*, coagulase-negative *Staphylococcus*, *Bacillus* spp., *Acinetobacter* spp.	N/A	2021	[115]
	Outdoor air	*Pseudomonas* spp., *Staphylococcus* spp.	N/A		[115]
KSA	Antimicrobial resistance genes in water bacteria	N/A	*mcr-1* gene	2024	[116]
Tunisia	Waste water–sewage treatment	*E. coli*	*mcr-1* gene	2020	[5]
	Raw water	*K. pneumoniae*	N/A		[5]
		*E. cloacae complex*	N/A		[5]
Lebanon	Irrigation/domestic/sewer waters in Syrian refugee camps	*E. coli*	*mcr-1*, *mcr-1.1* gene	2019–2021	[117,120]
	Domestic/sewer waters in Syrian refugee camps	*P. mirabilis*	*mcr-1* gene	2020	[121]
	Rainbow trout aquaculture	*E. coli*	*mcr-1* gene	2020	[99]

MDR: Multi-Drug resistant; ESBL: Extended-Spectrum Beta-Lactamase; *E. coli*: *Escherichia coli*; *K. pneumoniae*: *Klebsiella pneumoniae*; *A. baumannii*: *Acinetobacter baumannii*; *E. cloacae*: *Enterobacter cloacae*; *P. mirabilis*: *Proteus mirabilis*; ST: Sequence Type; mcr: mobile colistin resistance; N/A: Not applicable; KSA: Kingdom of Saudi Arabia.

## Data Availability

No new data were created or analyzed in this study. Data sharing is not applicable to this article.

## Abbreviations

The following abbreviations were used in the paper:

MENA	Middle East and North Africa
GNB	Gram-negative bacteria
LPS	Lipopolysaccharide
ESBL	Extended Spectrum Beta-Lactamase
MCR	Mobile Colistin Resistance
PEtN	Phosphoethanolamine transferase
CAMPs	Cationic antimicrobial peptides
Pag	Polymyxin-activated genes
TCS	Two-component system
RTE	Ready-to-eat
PFGE	Pulsed-field gel electrophoresis
ICU	Intensive care unit
CRKP	Carbapenem-resistant *Klebsiella pneumoniae*
ExPEC	Extra-intestinal pathogenic *Escherichia coli*
SARS-CoV-2	Severe acute respiratory syndrome coronavirus 2
MIC	Minimum Inhibitory Concentration
WGS	Whole genome sequencing
ST	Sequence Type
CC	Clonal Complex
ATB	Antibiotic
AST	Antimicrobial susceptibility testing
AMX	Amoxicillin
AMC	Amoxicillin-clavulanic acid
SXT	Trimethoprim-sulfamethoxazole
